# Supplementary material for: Squirming motion of baby skyrmions in nematic fluids
Source: Nat Commun. 2017 Sep 22;8:673. doi: 10.1038/s41467-017-00659-5 (PMC5610258; doi:10.1038/s41467-017-00659-5)
Supplement: Supplementary file 1 — Supplementary Information [file 41467_2017_659_MOESM1_ESM.pdf]

Description of Supplementary Files

File Name: Supplementary Information

Description: Supplementary figure, supplementary references

File Name: Supplementary Movie 1

Description: Reversible motion of a baby skyrmion

File Name: Supplementary Movie 2

Description: POM textural changes of the baby skyrmion during the directional swimming

Supplementary Figure

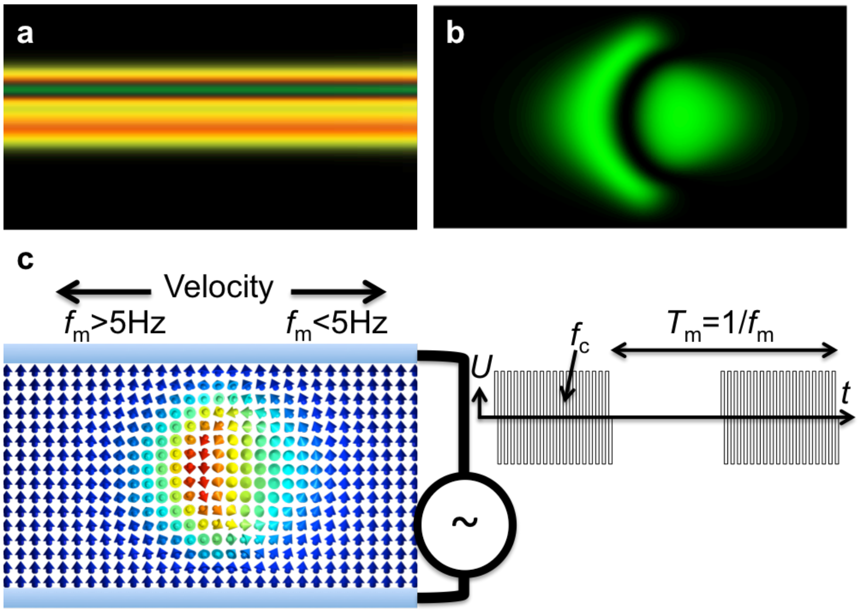

Supplementary Figure 1 | Baby skyrmion oriented orthogonally to $\mathbf{n_0}$. (**a**) Computer-simulated analog of the POM image shown in Fig. 1g. (**b**) Computer-simulated analog of the 3PEF-PM image shown in Fig. 1h. These **n**(**r**)-structure and images correspond to one type of cholesteric fingers that we studied previously.[1,2] (**c**) A schematic showing **n**(**r**) and geometry of a sample in which translational motion of a baby skyrmion is induced in one of the two directions orthogonal to it, depending on f_m.

(1) Zhang, Q., Ackerman, P. J., Liu, Q., & Smalyukh, I. I., Ferromagnetic switching of knotted vector fields in liquid crystal colloids. *Phys. Rev. Lett.* 115, 097802 (2015).

(2) Smalyukh, I. I., Senyuk, B., Palffy-Muhoray, P., Lavrentovich, O., Huang, H., Gartland, E., Bodnar, V., Kosa, T., Taheri, B., Electric-field-induced nematic-cholesteric transition and three-dimensional director structures in homeotropic cells. *Phys. Rev. E* **72**, 061707 (2005).
